# Fabrication and Physicochemical Investigation of Ancient Iranian and Pakistani Treated Silver Particles and their Comparison with Silver Nanoparticles

**Published:** 2017

**Authors:** Soheyla Honary, Alireza Mozaffari Dehshiri, Mahmoud Mosaddegh

**Affiliations:** a *Department of Pharmaceutics, School of pharmacy, Mazandaran University of Medical Sciences, Sari, Iran.*; b *Department of Traditional Pharmacy, School of Traditional Medicine, Shahid Beheshti University of Medical sciences, Tehran, Iran.*

**Keywords:** Pakistani silver Kushtas, Iranian Silver Kushtas, Silver Nanoparticles, Iranian Traditional Medicine (ITM)

## Abstract

Silver compounds are known to be both toxic and carcinogenic. However, silver nanoparticles have been showed diagnostic and therapeutic value. They can be used as biological tags for a quantitative detection and/or be incorporated into wound dressings and cosmetics due to their antibacterial properties. Pakistani and Iranian traditional physicians still take the advantage of silver compounds, called silver Kushta, to treat dementia, leprosy, and skin cancers. The present study compared the physicochemical properties of silver nanoparticles (AgNPs) and silver Kushtas (Pakistani silver Kushta (PKAg) and Iranian silver) in terms of the morphology, silver content, chemical composition, and suspension stability. AgNPs were produced through a chemical reduction method using AgNO_3_ and NaBH_4_ at 4 °C. PKAg powder was purchased from Hamdard pharmaceutical company (Pakistan). IKAg powder was produced, using closed reactor (Aghili method). Physicochemical properties of all three compounds were examined by scanning electron microscopy, dynamic light scattering, Fourier transform infrared, UV spectra Analysis, Energy Dispersive X-ray analysis, and X-ray diffraction. AgNPs were spherical-shaped and uniform in size. However, PKAg and IKAg particles show different sizes. Not only AgNPs but also IKAg & PKAg Particle sizes were less than 200 nm. According to EDX analysis, the silver contents of PKAg, IKAg, and were 66.24%, 50.43%, respectively. AgNPs, IKAg, and PKAg showed zeta potential values equal to -18.5 and -2.27, and -12 mV, successively. AgNPs, IKAg and PKAg sizes were 64.08, 190.4 and 51.72 nm, respectively. Moreover, XRD results indicated that the composition of IKAg and PKAg are completely different.

## Introduction

*Aim of the study*

This study aimed to answer the following questions:

- What is the standard operating procedure (SOP) to produce Iranian silver Kushta (IKAg), currently an abolished dosage form, according to ancient references?

- Is there any relationship between these kinds of old dosage forms and nanotechnology? (Were they the first generation of nanoparticles?)

- What are the physicochemical properties of Pakistani silver Kushta (PKAg), widely used as traditional medicine in various countries, compared to silver nanoparticles (AgNPs)?

We also sought to produce IKAg and compare its physicochemical properties, e.g. size, with PKAg and AgNPs.

*Background/Context: Silver Kushta and Nanosilver*

Based on the collected data, one out of three people, especially in Asian and African countries such as Iran and Egypt are interested in using the alternative medicines (1, 2). These people believe that using traditional medicines can help them to meet some of their healthcare needs. People in developed countries are not an exception, since 75% of Germans, 70% of Canadians, and 47% of the British population tend to regularly use some forms of traditional medicines (3). The globalization of traditional medicines has an important implication for quality control and standardization criteria (4). Kushtas are among the most significant preparations in Unani and Ayurvedic medicine. 

The word “Kushta” is believed to have been derived from the Persian word “kushtan” (meaning “to kill”). Kushtas are medicinal powders obtained from the calcination of metals, minerals as well as animal and herbal extracts. They are thus mixtures of metal and nonmetal oxides (5, 6) produced in Iranian and Ayurvedic medicine by heating and calcination of metals to kill (omit) the toxicity of heavy metals (6, 7). Adding herbal extracts to this process is also thought to promote the quality of the final product (5-7). Avicenna, an Iranian scholar, introduced nearly 50 kinds of Kushtas in his valuable book Qanoon (7, 8).

Although nanosilver is a novel topic in the modern pharmacy, ancient and medieval pharmacopeias such as Qanoon of Avicenna (6-9), Qarabadin of Aghili, and Alhavi of Rhazuz seem to have argued its usages as a treatment for melancholy, bronchitis, dementia, leprosy, Alzheimer’s and heart diseases (10, 11). They have also explained some key-concepts regarding the production of such compounds using incineration in high temperature. Hakims (physicians) of Iranian traditional medicine believed that calcination (Altaklis) resulted in fine particles which could be easily absorbed (12, 13). They prescribed silver Kushtas in combination with “*borage dough*” (*khamireh Gaozaban*) and (*Jawaharwala*) for palpitation (tachycardia) (5). Calcination, a severe heating process, used to change the quality of materials in order to reach the fine powders. According to Iranian medical manuscript, calcination can be performed (5, 13) in the constant temperature of 960 °C for 20 min. Seyyed Mohammad Hussain Aghili Khorasani, a famous Iranian Hakim, whose books are widely used in both Iranian and Indian traditional medicine, dedicated a chapter in his book (Qarabadin) to Kushtas (10, 14)*. *Although pharmacological study on the therapeutic effects of Kushtas was performed (5), no surveys have particularly focused on IKAg yet. 

The advantages of nanosilver, e.g. its anti-bacterial, anti-cancer, and antitumor effects (15, 16), make it an interesting subject in the field of nanomedicine. Colloidal dispersion is usually utilized for systems in which the particle size of the dispersed phase ranged between 1 and 1000 nm and the dispersion medium is a liquid (17-19). A series of certain drugs show therapeutic effects when formulated in a colloid state such as silver nanoparticles. Despite the existence of various methods to prepare nanosilver colloids, using chemical reduction is the most broadly used. In this study, according to the previous literatures, sodium borohydride (NaBH_4_) used as a reductant in the chemical processes to produce AgNPs from silver nitrate (AgNO_3_) solution (20, 21). 

The size of AgNPs in this process depends on various factors such as stirring rate, and temperature (22). The stability of many colloidal systems is closely related to the magnitude of zeta potential. Generally, colloidal systems with sufficiently high absolute quantity of zeta potential are stable. Conversely, the system agglomerates if its zeta potential was closed to zero (23). 

The present study aimed to prepare IKAg according to Iranian traditional medicine (ITM) and to compare its physicochemical properties, such as silver content, size and shape with commercially available (PKAg) and chemically facilitated AgNPs.

## Experimental

*Materials*

PKAg was purchased from Hamdard Co. Pakistan. Sodium chloride, AgNO_3_, and NaBH_4_ were all purchased from Merck, Germany. Silver powder was bought from Sigma-Aldrich, USA. High molecular weight (MW) chitosan were purchased from Fluka (Switzerland).

*Preparation of IKAg*

Silver Kushtas and AgNPs were produced through calcination procedure and chemical reduction method using AgNO_3_ and NaBH_4_ as a chemical reducer. To obtain modified IKAg the process was conducted based on Aghili’s manuscripts, Qarabadin and Makhzan Al-Adviyah (10, 14). Totally, 8 g silver fine powder and 750 mg of sulfur were added to 135 mL of 10% sodium chloride solution in a dry round-bottomed closed cylindrical tin worked stainless steel container. The apparatus then heated at 960 °C for 20 min. Finally, the obtained component was pounded and grinded in a pestle and mortar. 

*Preparation of AgNP dispersion*

AgNPs were obtained by chemical reduction method (with NaBH_4_ as the reductant) at 4 °C. A stock solution of chitosan (2 mg/mL) was prepared by dissolving 5 mg chitosan in 10 mL of 10% solution of acetic acid (1% V/V). Due to low solubility of chitosan, the mixture was vibrated on a magnetic stirring instrument and maintained at room temperature (25 °C) for 24 h. The solution was filtered through a 0.22 μM Millipore filter to prevent any impurity before application. Complete reduction was assured by considering the concentration of NaBH_4_ (0.2 mol/L) to be 10 times greater than that of AgNO_3_ (0.02 mol/L) (24). The colloidal silver nanoparticles reveal brown black color.

*Kushtas’ suspensions preparation and their specifications*

A sieve with pore mesh size of 200 was used during the process to separate the fine Kushta powder. 250 mg of each triturated Kushta powder (PKAg and IKAg) were added in sequence to a beaker containing 25 mL distilled water to prepare Kushta suspensions. The solutions were then homogenized by three 10-minute intervals of ultra-sonication with two-minute lag times (sonic power density: 600 watt/m^2^). The beaker of sample was placed in ice-bath during the process. IKAg suspension reveals dark black but PKAg is whitish-gray color.

*Kushtas’ suspendability*

The colloidal dispersion of both Kushtas was performed by suspending 5 g of silver Kushta in 50 mL distilled water using a magnetic stirrer followed by 15 min of continuous ultra-sonication. (Sonic power density: 600 watt/m^2^). In order to determine the suspendability of Kushtas, both Kushtas dispersions were separated from solid sediments. The sediments then placed in an oven at 80 ºC for one hour to obtain the dried weight for each sample. The quantity of dispersion phase for each sample calculated by following formula:

*Equation 1: C (mg/mL) = (*M-m/V*)×1000*

In this equation, M stands for: amount of silver component in suspension *m* stands for amount of sediment of silver component after sedimentation and drying in oven at 80 °C and V stands for volume of water used in the suspension as a continues phase.

*EDX and silver rates in Kushtas*

In this study, Energy Dispersive X-ray spectroscopy was used as an analysis method to detect elemental silver. The samples were dried at room temperature and then analyzed for sample composition. EDX was carried out under a Rontec (USA). The detector was TESCAN model VEGA 2 LM (Check republic). The method was used to show the presence of silver and different elements and their percentages (25, 26). 

*Morphological properties of silver component using SEM technique*

Scanning electron microscope model XL 30 Philips (Netherland) was used to analyze the surface and shape characteristics of the particles after they were coated with gold. 

*Size and zeta potential measurements of AgNPs and silver Kushtas*

The measurement of particle size and the stability of PKAg, IKAg and AgNPs in all samples were done using Malvern zetasizer. Poly disparity and zeta potential were determined by a zeta analyzer using zetasizer 3600 at 25 °C with a scattering angle of 90 ° (Malvern Instruments Ltd, UK).

*UV visible studies*

The reduction of silver metal ions to silver nano particle was analyzed by using UV-visible spectrometer (Perkin – Elmer, Lambda 25 USA) between wavelengths of 270- 530 nm. UV-visible spectroscopy is a useful method to determine the formation of metal nanoparticles in an aqueous solution. The λ-max in solution does arise because of the excitation of surface plasmon vibrations in the silver nanoparticles. The strong surface plasmon resonance bond appears at the range of 400-500 nm (26-28).

*X-ray diffraction (XRD) studies and the reflection lines of kushtas*

XRD is a rapid analytical technique primarily used for phase identification of crystalline materials. Analysis of chemical compound structures can provide information on unit cell dimension. The XRD patterns of PKAg and IKAg were recorded on an X-ray diffractometer using radiations with λ = 1.5406 Å filtered by nickel foil over a temperature range of 5-110 °C, for IKAg and PKAg. Assuming that the crystallites were free of non-uniform strains, the crystalline domain size was calculated from the width of XRD peaks using the Scherer formula: 

*Equation 2: D = ĸλ/βcosθ*

where *ĸ *is Scherer constant (= 0.94), *λ *is the X-ray wavelength, *β *is the half maximum of the peak width, and *θ *is the Bragg diffraction angle (26-29).

Powder X-ray diffraction pattern recorded by using the X-ray diffractometer (model XPERT-PRO) and software (XPERT-High score), PANalytical (Netherland) was used to show IKAg and PKAg component.

*FTIR spectroscopy*

FTIR studies carried out to determine different chemical functional groups such as hydroxyl and sulfide. KBr discs were prepared for FTIR spectroscopy using FTIR instrument (PerkinElmer 1320 FTIR spectrophotometer, USA). The results of FTIR spectroscopy, with 16 scans/ minute, were recorded on a Bruker Tensor system. The chemical compositions of AgNPs and Kushtas were ultimately determined by computer software.

*Physical stability of silver Kushtas and AgNPs*

The physical stability of suspensions carried out by comparison between particle sizes determined by the Malvern Zetasizer (Malvern Instruments Ltd, UK) before and after a period of nine months. 

## Results and Discussion

*The quantity of dispersion phase in Kushtas suspensions*

Calculation shows the quantity of dispersion phase in PKAg suspension (4.61 mg/mL) is more than IKAg suspension (4.2 mg/mL), based on the formula discussed in section 2-2-4. 

*Silver rates in Kushtas using EDX spectrometry method*

**Figure 1:**
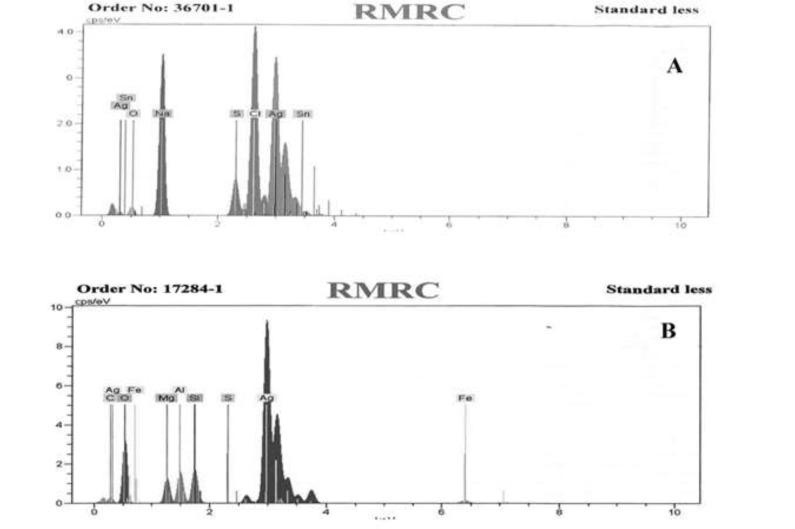
EDX spectra of IKAg (A) and PKAg (B).

PKAg and IKAg were analyzed through EDX spectrometer to determine their components. The results show that PKAg consisted of 66.24% silver, 3.42% aluminum, 3.38% silicon, 2.89% magnesium, 1.87% iron, 0.76% carbon, 0.14% sulfur, and 21.29% oxygen. However, IKAg contained 50.43% silver, 2.15% oxygen, 21.93% sodium, 3.6% sulfur, 20.3% chloride and 1.59% tin (Figure 1). 

*Morphological properties using SEM technique*

**Figure 2:**
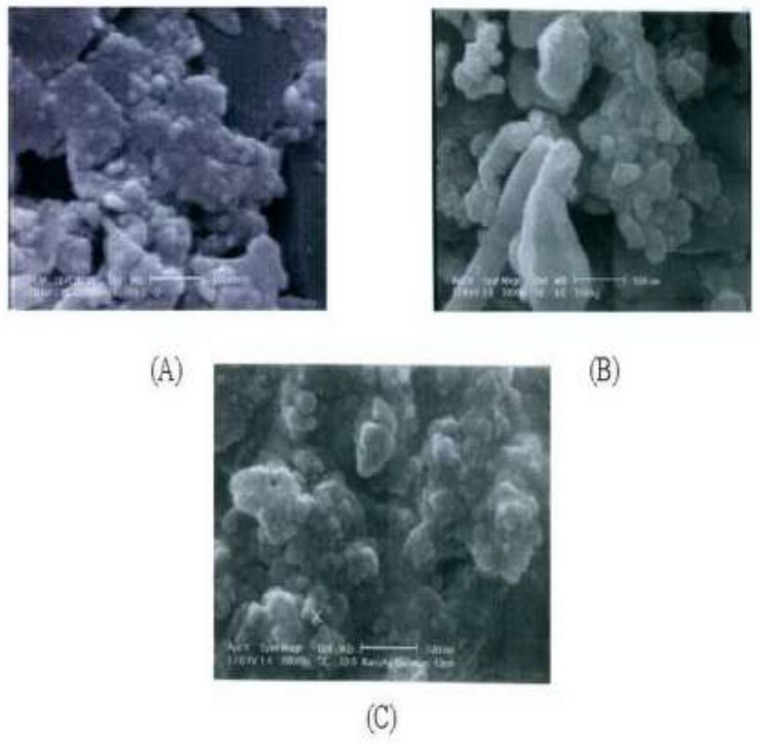
SEM micrographs of the IKAg (A) and PKAg (B) and AgNPs (C).

According to the SEM results, there is a size distribution for each of three silver samples ranging from 25 to 200 nm for PKAg, 53 to 426 nm for IKAg, and 43 to 298.8 nm for AgNPs. Figure 2 clearly illustrates that although AgNPs show almost spherical shape, Kushtas have both spherical and irregular shapes. However, the shape diversity in IKAg is more than others. Both kinds of silver Kushtas show two proportions of particles; one portion smaller than 10 nm and the other bigger than 10 nm. Our findings also show that both of the Kushtas could be categorized as kinds of old nanoparticles. However, they have a proportion of large particles too, which were separated during the suspension preparation process. Among the samples, IKAg, with an intensity mean diameter of 190.4 nm, shows the biggest size. On the other hand, SEM results showed that AgNPs and PKAg were similar in terms of not only particle size (e.g., 78.6 and 50.79 nm), but also geometrical shapes. Besides, SEM results illustrated that both silver Kushtas show different shapes such as spherical, rock and plate forms. However, the diversity of sizes and shapes is more evident in IKAg compared to that of PKAg and there is more similarity in terms of shape between PKAg and AgNPs owing to a higher proportion of spherical particles in both samples. Totally, we can say that the SEM micrographs confirmed the previous DLS results.

**Figure 3:**
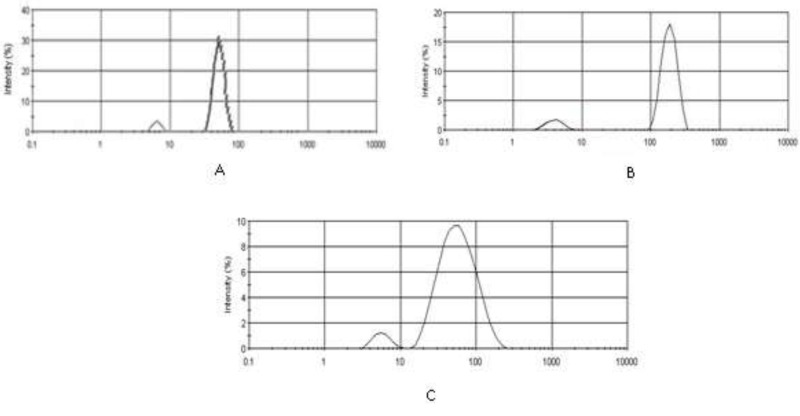
Polydispersity index (PDI) and Intensity mean diameters for (A) IKAg = 0.8, d = 190.4 nm, (B) PKAg = 0.9, d = 51.72 nm, and (C) AgNPs = 0.22, d = 64.08 nm

**Figure 4.**
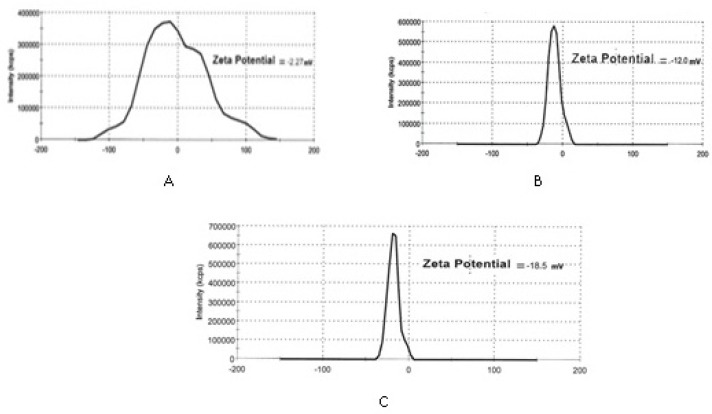
Zeta potential of IKAg (A), PKAg (B) and AgNPs (C).

**Figure 5:**
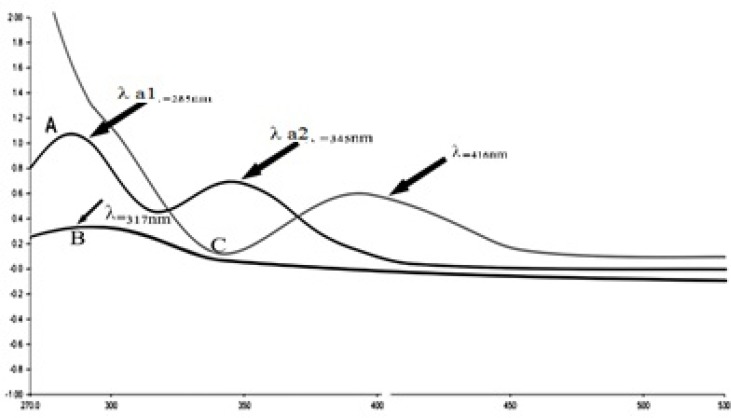
UV-Visible spectra of IKAg (A): λ a1 = 285 nm and λ a2 = 345 nm, PKAg (B): λ = 317 nm and AgNPs (C): λ = 416 nm

**Figure 6:**
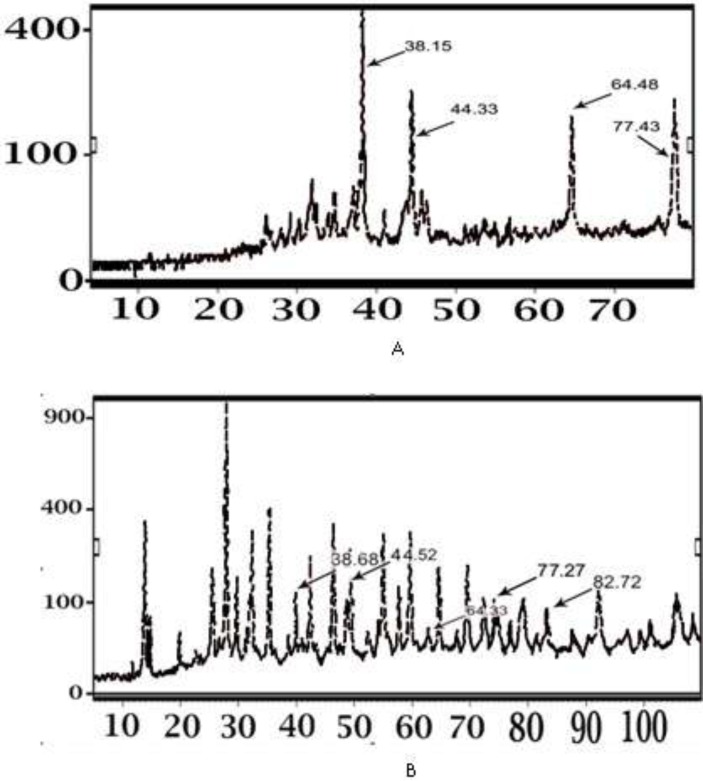
X-ray diffraction pattern of IKAg (A) and PKAg (B) at room temperature

**Figure 7:**
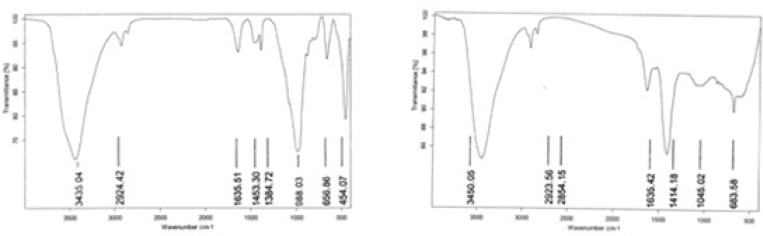
FTIR spectra of IKAg (A) and PKAg (B) at a region of 500-3500 cm^-1^.

**Figure 8:**
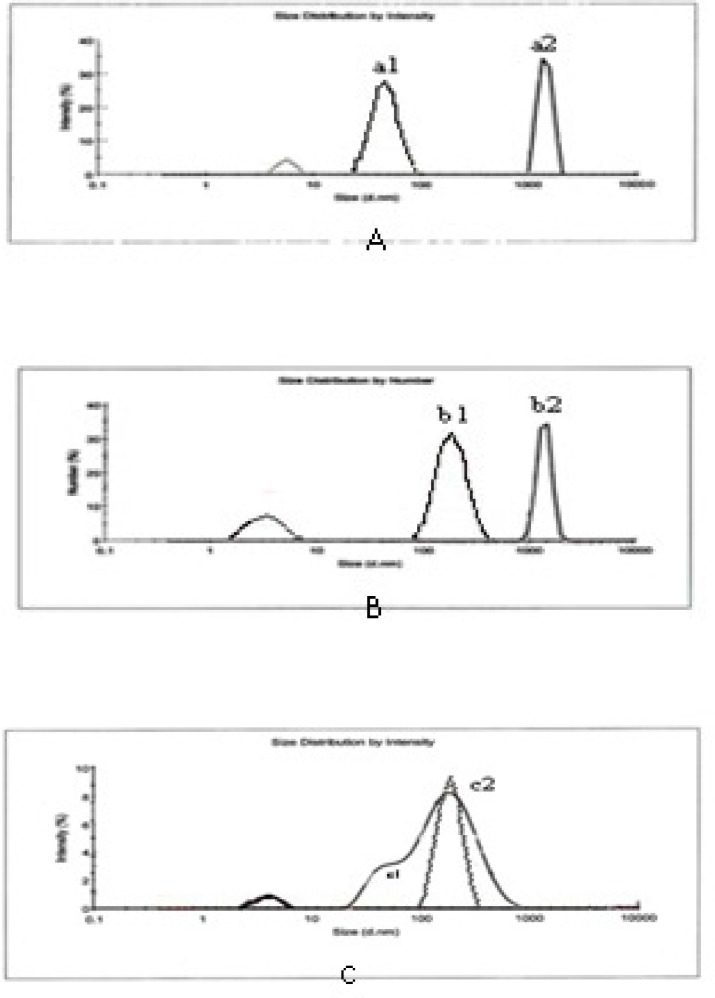
Stability of silver Kushta suspension [IKAg (A), PKAg (B) and AgNPs colloid system (C)]. a1, b1, c1 show the first curves and a2, b2, c2 show the second curves of silver component

*Particle size characterization and zeta potential measurements*

Figures 3 and 4 depict particle sizes and zeta potential of AgNPs, PKAg, and IKAg. The intensity mean diameters of samples are 64.08, 51.72 and 190.4 nm for AgNPs, PKAg and IKAg, respectively. Besides, the zeta potential values are equal to -18.5, -12 and -2.27 mV for PKAg, IKAg and AgNPs, successively. Figure 3 also clearly shows the peaks states and the size C for each suspension. 

*Physical properties and UV-visible spectra analysis*

To confirm the presence of nano sized particles, a UV-Vis method was carried out. A color change from brown to dark gray occurs when Ag colloidal nanoparticles were fabricated in a solution. This change is attributed to the surface Plasmon resonance (SPR) phenomenon (32). UV visible spectroscopy is a helpful method to manifest the size of colloidal particles owing to SPR and optical absorption spectra of metal nanoparticles which can alter longer wavelengths as particle size increases (20). To make a long story short, we can simply say that the existence of a λ max under 500 nm confirms the presence of nano sized particles in the sample.

IKAg, PKAg, and AgNPs dispersions manifested as dark black, whitish-gray and gray black suspensions. The prepared AgNPs and silver Kushtas colloids were periodically scanned from 270–530 nm to assess the absorption spectra for each sample. The λ-max for each sample under 500 nm confirms the presence of silver nanoparticles owing to the exhibiting characteristic surface Plasmon resonance peak. The obtained absorption spectra match with result of previous literatures (26-28). The results indicate that PKAg, IKAg and AgNPs showed absorption band at 317, 345 and 416 nm, respectively (Figure 5). 

*XRD studies*

Figure 6 shows the X-ray diffraction pattern obtained from PKAg and IKAg. The XRD clearly indicates that the silver particles are crystalline in nature. Besides, the diffraction pattern of IKAg closely matched with the standard diffraction of silver formed in AgNPs in the previous literatures (26, 29). The four diffraction peaks at [2θ = 38, 44, 64 and 77] were indexed as (111), (200), (220), (311) planes of face-centered cubic (FCC) silver according to the “Expert high score” software. The x-ray diffraction pattern of IKAg completely matched with this reference pattern (Figure. 6). However, the diffraction pattern of PKAg showed that there are some other components such as calcium sulfate (CaSO_4_), AgCl, SiO_2_, Fe_2_O_3_ in this kind of Kushta (Figure.6) based on the aforementioned software. 

Our EDX results showed that the percentages of silver in both Kushtas are similar to each other, 50.43% in IKAg and 66.24% in PKAg. However, they show differences in terms of having other ingredients such as aluminum, silicon, magnesium and iron, which are just present in PKAg. Regarding the results provided by EDX, it was found that the main elements of PKAg and IKAg are Ag, oxygen, Fe, Si and Ag, Na, Cl, S and O, respectively. The difference between Kushtas in terms of ingredients and crystallinity was assessed by using other methods such as XRD and FTIR. The results confirmed that while silver constituted the major part of both PKAg and IKAg, other elements differed in each sample. According to the XRD results, IKAg consisted of Ag2S, Ag2O3, Ag2O, Ag3C, Ag, and CS2. However, calcinated PKAg included CaSO4, AgCl, SiO2, Fe2O3 and Na2O. Moreover, XRD results ascertained the presence of CaSO4 and Ag2S in PKAg and IKAg, respectively. Finally, the successive peaks at 683 and 655 nm in FTIR analysis for PKAg and IKAg, indicate the presence of C—CL groups, which may be caused by using sodium chloride in the production processes of both Kushtas. Probably differences in calcinations method or employing herbal extracts such as *Aloe Vera* may have affecte

d the chemical compositions for each Kushta.

*FTIR studies*

FTIR spectroscopy of both silver Kushtas, Figure 7, showed different chemical functional groups at the region between 450 and 3500 cm^-1^. The important detected peaks are at 3450, 2923, 1414, 1045 and 683 cm^-1^ for PKAg and, 3435, 2924, 1453, 988, 656 cm^-1^ for IKAg. The observed peaks in both Kushtas showed vibration bands at 3450 and 3435 cm^-1^ for PKAg and IKAg, respectively. These peaks could be related to either OH or NH_2_, owing to their overlapping in this region (27, 30). The peaks at 2923 and 2924 for PKAg and IKAg, successively, can be assigned as absorption band of –C=H stretching vibration. Besides, rising peaks at 1414 and 1453 cm^-1^ for PKAg and 1453 for IKAg indicate the probable presence of sulfate and sulfide groups (27, 30). Nevertheless, peaks at 1045 cm^-1^ for PKAg and 988 cm^-1^ for IKAg suggest the presence of C-O stretching group (27, 30). Finally, peaks at 683 for PKAg and 655 cm^-1 ^for IKAg confirm the presence of C-CL group (27, 30) (Figure. 7). 

*Stability evaluation of silver Kushtas and AgNPs*

For stability evaluation tests for silver Kushta suspensions and AgNPs colloids were performed 4 °C for 9 months (31). Sample sizes were measured before and after the period of time (9 months). The experiments showed that PKAg sizes increased only 3.3 times after nine months However IKAg sizes increased about 6.9 times during the timescale and AgNPs sizes increased by almost two times (from 64.08 to 134 nm) (Figure 8). The first curve shows silver component particle sizes in primary stages and the second curves implies sizes of the particles after 9 months.

Physical stability is one of the most important characters that a nanosuspension should have. The lack of sufficient physical stability causes particle aggregation and produces bigger particles. Totally, the higher the value of zeta potential and the more the uniformity of particle size, the more the physical stability of the system (33, 34). According to the stability results, AgNPs showed the best physical stability with a size increase of 2 times followed by PKAg (3.3 times). The worst sample in terms of stability is IKAg with 6.9 times size increase. Moreover, zeta potentials for AgNPs and two silver Kushta suspensions, PKAg and IKAg, were -18.5, -12 and -2 mV, respectively. Totally, to stabilize a nano system, a zeta potential of ± 30 mV is required (33, 34). Besides, previous literatures reported several ways in order to increase the physical stability of AgNPs. Most of these methods were based on using polymers such as glycosaminoglycans, polyvinylpyrrolidone (PVP) and chitosan in order to coat the nanoparticles and enhance their physical stability (31, 35, 36). These materials play the role of a stabilizer and prevent particles from aggregation over the time of storage (37, 38). Overall, in our samples physical stability provided with using two main factors: zeta potential and stabilizers. Among the samples, AgNPs have both of them (chitosan and ZP = -18.5 mV). On the other hand, for IKAg there is no stabilizer and the ZP close to zero. That is why the former and later showed the highest and lowest stabilities among the samples, respectively. Meanwhile, PKAg with ZP = -12 stood in the middle. Although the method of fabrication for PKAg is not clear, we know they use some animal and plant extracts in this process (5). It is not strange if some of these extracts have some proportion of polymers such as polysaccharides, which could play the role of a stabilizer in these systems. So, these kinds of Kushta are supposed to be more stable compared to the ones without extracts.

## Conclusion

The conclusion could be drawn from different aspects. First of all, we found that IKAg can be easily produced by using silver, NaCl, distilled water, and a bit of sulfur powder at temperatures above 960 °C in a closed reactor. However, the size of particles in this method is not as small as AgNPs and/or PKAg owing to the differences between the preparation methods. It seems that the process of calcination was repeated several times (almost seven times) for PKAg but in this study it just carried out once for IKAg. Totally, the more repetition of calcination, the more uniform and smaller the particles produce. Moreover, with the replication of calcination, the rate of Ag increases. So, the repetition of calcination process is strongly recommended for reaching smaller and more uniform nanoparticles. However, the temperature could be an effective factor too. 

On the other hand, using animal and herbal extracts during the production method could have a stabilizing role and affect the ZP which results in more stable nano particles in a suspension. However, using these kinds of ingredients could cause more problems in terms of presenting more impurities in the sample. 

Finally, we can undoubtedly categorize Kushtas as an ancient form of nanoparticles with more efficacies, owing to their higher purity and extremely small size. 

The evaluation of all kinds of traditional Kushtas may lead to the production of new therapeutic protocols to treat currently incurable diseases such as multiple sclerosis and cancer.

## References

[1] Galabuzi C, Agea J, Fungo B, Kamoga R (2010). Traditional medicine as an alternative form of health care system: a preliminary case study of Nangabo sub-county, central Uganda. Afr. J. Tradit. Complem..

[2] Kayne S (2009). Introduction to traditional medicine Complementary and Alternative Medicine.

[3] Bodeker G (2001). Lessons on integration from the developing worldʹs experience. BMJ.

[4] Keen R, Deacon A, Delves H, Moreton J, Frost P (1994). Indian herbal remedies for diabetes as a cause of lead poisoning. Postgrad. Med J..

[5] Vohora SB, Athar M (2008). Mineral drugs used in Ayurveda and Unani medicine.

[6] Sharafkandi A (2008). The Canon of Medicine of Avicenna.

[7] Sharafkandi A (2008). The Canon of Medicine of Avicenna.

[8] Sharafkandi A (2008). 7th ed. The Canon of Medicine of Avicenna.

[9] Sharafkandi A (2008). The Canon of Medicine of Avicenna.

[10] Aghili AKSS, Gharabadin kabir (2007). The Institue of Medical history, Islamic and complementary medicine studies.

[11] Tabatabaei SM (2011). A brief of Alhavi.

[12] Ryffel B, Di Padova F (2013). IL-17, IL-22 and Their Producing Cells: Role in Inflammation and Autoimmunity.

[13] Soleimani A, Sediqi MFT (2004). A survey of Drug.

[14] Aghili AKSS (2008). Makhzan ul-Advia.

[15] Momen Tonekaboni M (2008). Tohfat al Momenin.

[16] Sriram MI, Kanth SBM, Kalishwaralal K, Gurunathan S (2010). Antitumor activity of silver nanoparticles in Dalton’s lymphoma ascites tumor model. Int. J. Nanomedicine.

[17] Brown CL, Bushell G, Whitehouse MW, Agrawal D, Tupe S, Paknikar K, Tiekink ER (2007). Nanogold-pharmaceutics: (i) the use of colloidal gold to treat experimentallyindused arthritis in rat models; (ii) Characterization of the gold in Swarna bhasma, a microparticulate used in traditional Indian medicine. Gold Bulletin..

[18] Anderson BD, Fox JL, A. Martin, J. Swarbrick, A. Cammarata (1985). Physical pharmacy: Physical chemical principles in the pharmaceutical sciences. PA. J. Pharm. Sci..

[19] Allen L, Popovich NG, Ansel H (2002). Pharmaceutical dosage forms and drug delivery systems.

[20] Michaels AM, Nirmal M, Brus L (1999). Surface enhanced Raman spectroscopy of individual rhodamine 6G molecules on large Ag nanocrystals. J. Am. Chem. Soc..

[21] Dang JM, Leong KW (2006). Natural polymers for gene delivery and tissue engineering. Adv. Drug Deliv. Rev..

[22] Aihara N, Torigoe K, Esumi K (1998). Preparation and characterization of gold and silver nanoparticles in layered laponite suspensions. Langmuir.

[23] Borges O, Cordeiro-da-Silva A, Romeijn SG, Amidi M, de Sousa A, Borchard G, Junginger HE (2006). Uptake studies in rat Peyerʹs patches, cytotoxicity and release studies of alginate coated chitosan nanoparticles for mucosal vaccination. J. Control Release..

[24] Honary S, Barabadi H, Gharaei-Fathabad E, Naghibi F (2013). Green synthesis of silver nanoparticles induced by the fungus Penicillium citrinum. Trop. J. Pharm. Res..

[25] Dimitrijević R, Cvetković O, Miodragović Z, Simić M, Manojlović D, Jović V (2013). SEM/EDX and XRD characterization of silver nanocrystalline thin film prepared from organometallic solution precursor. J. Min. Metall B..

[26] Gnanajobitha G, Annadurai G, Kannan C (2012). Green synthesis of silver nanoparticle using Elettaria cardamomom and assesment of its antimicrobial activity. Int. J. Pharm. Sci. Drug Res..

[27] Geetha N, Harini K, Showmya JJ, Priya KS (2012). Biofabrication of Silver Nanoparticles Using Leaf Extract of Chromolaena Odorata (L.) King and Robinson.

[28] Jha AK, Prasad K (2011). Green fruit of chili (Capsicum annum L) synthesizes nano silver. Dig. J. Nanomater. Bios..

[29] Shah W, Patil U, Sharma A (2011). Green synthesis of silver nanoparticles from stem bark extract of Terminalia tomentosa Roxb (Wight & Arn). Der Pharma chemica..

[30] Socrates G, H.W. Siesler (2004). Infrared and Raman characteristic group frequencies: tables and charts, in Near-Infrared Spectroscopy: Principles, Instruments, Applications.

[31] Hettiarachchi M, Wickramarachchi P (2011). Synthesis of chitosan stabilized silver nanoparticles using gamma ray irradiation and characterization. Journal of Science of the University of Kelaniya Sri Lanka.

[32] Moores A, Goettmann F (2006). The plasmon band in noble metal nanoparticles: an introduction to theory and applications. New. J. Chem..

[33] Honary S, Zahir F (2013). Effect of zeta potential on the properties of nano-drug delivery systems-a review (Part 1). Trop. J. Pharm. Res..

[34] Honary S, Zahir F (2013). Effect of zeta potential on the properties of nano-drug delivery systems-a review (Part 2). Trop. J. Pharm. Res..

[35] Kemp MM, Kumar A, Mousa S, Park T-J, Ajayan P, Kubotera N, Mousa SA, Linhardt RJ (2009). Synthesis of gold and silver nanoparticles stabilized with glycosaminoglycans having distinctive biological activities. Biomacromolecules.

[36] Bilberg K, Hovgaard MB, Besenbacher F, Baatrup E (2011). In vivo toxicity of silver nanoparticles and silver ions in zebrafish (Danio rerio). J. Toxicol..

[37] Huang H, Yuan Q, Yang X (2004). Preparation and characterization of metal–chitosan nanocomposites. Colloid Surface B..

[38] Shanmugam S, Viswanathan B, Varadarajan T (2006). A novel single step chemical route for noble metal nanoparticles embedded organic–inorganic composite films. Mater. Chem..

